# Enhanced thermoelectric properties of self-assembling ZnO nanowire networks encapsulated in nonconductive polymers

**DOI:** 10.1038/s41598-023-48385-x

**Published:** 2023-11-29

**Authors:** Margarita Volkova, Raitis Sondors, Lasma Bugovecka, Artis Kons, Liga Avotina, Jana Andzane

**Affiliations:** 1 Institute of Chemical Physics, University of Latvia, Raina Blvd 19, Riga, 1586 Latvia; 2 Faculty of Chemistry, University of Latvia, Jelgavas Str. 1, Riga, 1004 Latvia

**Keywords:** Energy science and technology, Materials science, Nanoscience and technology, Physics

## Abstract

The near-room temperature thermoelectric properties of self-assembling ZnO nanowire networks before and after encapsulation in nonconductive polymers are studied. ZnO nanowire networks were synthesized via a two-step fabrication technique involving the deposition of metallic Zn networks by thermal evaporation, followed by thermal oxidation. Synthesized ZnO nanowire networks were encapsulated in polyvinyl alcohol (PVA) or commercially available epoxy adhesive. Comparison of electrical resistance and Seebeck coefficient of the ZnO nanowire networks before and after encapsulation showed a significant increase in the network's electrical conductivity accompanied by the increase of its Seebeck coefficient after the encapsulation. The thermoelectric power factor (PF) of the encapsulated ZnO nanowire networks exceeded the PF of bare ZnO networks by ~ 5 and ~ 185 times for PVA- and epoxy-encapsulated samples, respectively, reaching 0.85 μW m^−1^ K^−2^ and ZT ~ 3·10^–6^ at room temperature, which significantly exceeded the PF and ZT values for state-of-the-art non-conductive polymers based thermoelectric flexible films. Mechanisms underlying the improvement of the thermoelectrical properties of ZnO nanowire networks due to their encapsulation are discussed. In addition, encapsulated ZnO nanowire networks showed excellent stability during 100 repetitive bending cycles down to a 5 mm radius, which makes them perspective for the application in flexible thermoelectrics.

## Introduction

Zinc oxide (ZnO) is an n-type semiconductor with a direct band gap of 3.37 eV, good biocompatibility, and high mechanical, thermal, and chemical stability^1,2^. These properties make ZnO suitable for a wide range of applications in different areas, such as light emitters^3^, sensors^4–7^, photovoltaics^8^, photocatalysis^9^, field effect transistors^10^, power nanogenerators based on piezoelectric^11^ and thermoelectric^12,13^ effect. In most applications, ZnO is used in the form of nanowires or nanorods to obtain devices with enhanced properties due to the high surface-to-volume ratio of the active material^14,15^. ZnO nanowires can be synthesized by a variety of methods, including chemical vapour deposition^16^, thermal evaporation^17^, molecular beam epitaxy^18^, pulsed laser deposition^19^, hydrothermal synthesis^20^, atomic layer deposition (ALD)^21,22^, and thermal oxidation of Zn foil/thin films^23–28^ or pre-grown Zn nanowires^29–31^. Among these methods, thermal oxidation of Zn is one of the most favorable as it does not require high-tech equipment, harmful chemicals, or very high synthesis temperatures and results in a high yield of ZnO nanowires. Typically, the process of thermal oxidation of Zn occurs in the air or oxygen at temperatures ranging from 300 to 600 °C^23–31^. However, this method's disadvantage is the difficulty separating the synthesized ZnO nanowires from the source material (Zn foil or Zn nanoparticle powder) to fabricate the ZnO nanowire network. The remains of the source Zn material can negatively affect the further performance of the ZnO nanowires.

In response to the increasing demand for affordable, effective, and non-toxic waste heat harvesting devices, ZnO-based rigid materials have been widely studied for their thermoelectric performance because of their natural abundance, low cost, and non-toxicity^32^. The efficiency of the thermoelectric materials is characterized by a dimensionless figure of merit *ZT* = *S*^*2*^*·σ·T·k*^*−1*^, where *S* is the Seebeck coefficient, *σ* is the electrical conductivity, T is the absolute temperature, and *k* is the thermal conductivity. (*S*^*2*^*·σ*) or (*S*^*2*^*·ρ*^*−1*^), where *ρ* is the electrical resistivity, is often referred to as the material's thermoelectric power factor (PF). Thus, a high Seebeck coefficient, which depends on charge carrier concentration^33^, is crucial for increasing ZT of thermoelectric material along with reducing *k* without affecting *σ*. To date, most of the research on the ZnO thermoelectric properties has focused on investigating ZnO thin films or ZnO powders-based ceramics^34–37^. Reported for these materials, Seebeck coefficients (*S*) were generally measured at elevated temperatures of 500–1000 °C and ranged from ~ − 100 to ~ − 380 µV K^−1^^35^, with some reports claiming *S* of ZnO powders-based densified ceramics up to ~ − 600 µV K^−1^ measured at room-temperature^34^. Knowledge of thermoelectric properties of ZnO nanowires or nanowire networks is limited to a few reports. Zappa et al.^12^ reported room-temperature S values of obtained by thermal evaporation ZnO nanowires ranging from ~ 110 to – 190 µV K^−1^, which correlate with the theoretical S values (from − 120 to − 150 µV K^−1^ at temperatures 300–600 K)^38^.

Despite the importance of the development of flexible thermoelectric materials application in wearable devices and waste heat harvesting from curved surfaces^39^, there is a minimal number of reports on the application of ZnO for flexible thermoelectrics at near-room temperatures^22,40^, where ZnO is used in the form of powder filler for electrically conductive polymer^40^ or deposited in the form of thin film on a flexible substrate^22^. Most state-of-the-art flexible thermoelectric materials developed for low-grade heat-to-power conversion are polymer-based composites with different nanostructured fillers, such as carbon nanotubes, bismuth and antimony chalcogenides, or hybrid fillers^41–45^. Commonly, the matrix for the polymer-based composite is electrically conductive polymers. However, despite the satisfactory efficiency of the electrically conductive polymers-based thermoelectric composites, reaching ZT up to 0.4^43^, the use of electrically conductive polymers as a matrix has some significant drawbacks. Mostly, these polymers have p-type conductivity and, thus, are more suitable for developing p-type composites rather than n-type. In turn, n-type electrically conductive polymers have poor stability in air and moisture, resulting in a significant decrease in their conductivity^46^. In addition, the poor processability of many electrically conductive polymers significantly complicates the synthesis of the composites.

Recently, our group demonstrated a novel approach for fabricating flexible n-type thermoelectric thin films by encapsulating nanostructured Bi_2_Se_3_-MWCNT and Sb_2_Te_3_-MWCNT hybrid networks in non-conductive polymers and application of these networks in flexible thermoelectric generators^47–49^. However, to date, the research of non-conductive polymer- or epoxy-based composites containing nanostructured ZnO filler was focused on the investigation of their mechanical, thermal, electrical, and optical properties for applications not related to thermoelectrics^50–53^.

In this work, self-assembling ZnO nanowire networks were synthesized by thermal oxidation of metallic Zn nanowire network pre-deposited on a solid (glass) or flexible (mica) substrate using low-pressure thermal evaporation. After oxidation, ZnO nanowire networks were encapsulated in PVA or commercially available epoxy adhesive using a drop-casting technique. The electrical and thermoelectric properties of the ZnO nanowire networks before and after encapsulation were studied at room temperature and compared. In addition, temperature-dependent thermoelectric properties of encapsulated in PVA and epoxy adhesive ZnO networks were studied in the target temperature range 180–380 K and compared. The encapsulated ZnO nanowire networks' bending behavior and mechanical and electrical stability were evaluated. To our knowledge, the thermoelectrical properties of PVA- and epoxy-encapsulated ZnO nanowire networks were investigated for the first time.

## Materials and methods

### Deposition of metallic Zn nanowire networks

Zinc foil (99.95%, thickness 50 µm, Goodfellow GmbH, Hamburg, Germany) was cut in pieces of approx. 2.5 × 6.0 cm (weight 0.22 g ± 0.02 g) and used as Zn source material for the deposition of Zn nanowire network. Before the synthesis, the Zn foil and glass substrates (Avantor, Radnor, PA, USA) were cleaned in acetone and isopropanol and dried under nitrogen gas (N_2_) flow. For the fabrication of flexible samples, freshly cleaved mica sheets (~ 60 µm thick, Goodfellow, Huntingdon, UK) Pre-cleaned Zn foil were placed into the center of the horizontal single-zone quartz furnace tube (OTF-1200X, MTI Corp., Richmond, CA, USA). The substrates were placed into the furnace tube in the area where the maximum temperature during the process will reach 300–350 °C. Before the synthesis, the quartz tube was flushed for a few minutes with N_2,_ pumped down to a pressure of 0.2 Torr, and sealed. For the evaporation of Zn source material, the furnace was heated up to 450 °C in the centre of the tube at a rate of 15°C min^−1^ and kept at this temperature for the next 60 min, followed by natural cooling down to room temperature.

### Oxidation of Zn nanowire networks to ZnO

Substrates with the pre-deposited Zn nanowire networks were placed in the centre of open to the ambient air tube of the furnace tube (OTF-1200X, MTI Corp., Richmond, CA, USA). The furnace was heated to 500 °C at a rate of ~ 16°C min^−1^ and kept at this temperature for the next 60 min, followed by natural cooling to room temperature.

### Encapsulation of ZnO nanowire network with PVA and epoxy adhesive

For the encapsulation in PVA, the 5 wt% solution of PVA (Celvol E 04/88S) and deionized water DIW) was ultrasonicated for 20 min and stirred for 30 min at a temperature of 70 °C. PVA-DIW solution was uniformly applied over the ZnO network and dried in air at 50 °C for 12 h. Epoxy adhesive (Henkel AG&Co.KGaA, Dusseldorf, Germany) was drop-casted on the surface of the ZnO nanowire network sample, followed by its spread throughout the network due to the capillary forces and dried under ambient conditions for 24 h. After encapsulation, the wt.% ratio of ZnO to encapsulating polymer was approximately 85:15.

### Morphological and structural characterization

The structure and morphology of prepared ZnO nanowire networks were inspected using a field-emission scanning electron microscope (SEM) Hitachi S-4800 (Hitachi Ltd., Tokyo, Japan). Powder X-ray diffraction (XRD) measurements were performed on a Bruker D8 Discover diffractometer (Bruker Corp., Billerica, MA, USA) using copper radiation source (Cu Kα = 1.54180 Å) with Bragg–Brentano geometry and a LynxEye (1D) detector. The divergence and anti-scattering slits were set at 0.6 mm and 8 mm, respectively. The patterns were recorded from 10° to 70° on the 2θ scale, using a scan speed of 0.5 s/0.02°. For the identification of the diffraction peaks, the ICDD database PDF-2/Release 2021 was used (Ref. cards PDF 01-078-9363 Zn and PDF 01-075-6445 ZnO). Fourier transform infrared (FTIR) spectra were measured with Bruker Vertex 70v spectrometer equipped with an attenuated total reflection module with diamond crystal. Measuring range 400–4000 cm^−1^, resolution ± 2 cm^−1^, 20 scans per spectrum. Measurements performed in vacuum, 2.95 hPa.

### Electrical and thermoelectric characterization

For electrical and thermoelectric measurements, electrical contacts (Au–Pd alloy, thickness 25 nm) were sputtered on the ZnO nanowire networks by ion beam coater Gatan 681 (Gatan, Inc., Pleasanton, CA, USA). The distance between the electrodes was 8–12 mm, and the sample width was 4–7 mm. Current–voltage curves of the sample were measured by a Keithley 6487 picoammeter/voltage source. Room-temperature Seebeck coefficient measurements were conducted under ambient conditions using a lab-made device reported elsewhere^48^ and calibrated by NIST Standard Reference material 3451 (NIST, Gaithersburg, MD, USA) for low-temperature Seebeck coefficient. Thermoelectric voltage, generated by the ZnO nanowire network, was registered by HP 34401A multimeter (Hewlett-Packard Company, Palo Alto, CA, USA) and custom software for automatic data recording. The temperature gradient between the sample sides did not exceed 5% from the absolute temperature at which the measurements were performed. The thermoelectric voltage generated by the samples before and after encapsulation was calculated as the mean of five consecutive measurements, and ± standard deviation [SD] was estimated from these five measurements. Measurements of temperature dependencies of the Seebeck coefficient, resistance, and thermal conductivity were performed under high vacuum conditions using the thermal transport option (TTO) of the physical property measurement system (PPMS DynaCool9T, Quantum Design, San Diego, CA, USA) in the temperature range from 180 to 380 K. The measurement errors were estimated by the PPMS software MultiVu by fitting different models to the experimental data.

### Bending tests

Two-point bending tests down to a 5 mm radius were performed using a custom experimental setup reported elsewhere^54^, allowing simultaneous bending of the sample and measuring its electrical current at a constant voltage applied to the sample. The resistance of the samples was calculated from applied to the sample voltage (0.1 V ± 1 mV) and measured current ([SD] ± 100 pA). For the data representation, the average resistance for three consecutive measurements was calculated, and [SD] was calculated using Excel for the Microsoft 365 function STDEV.S.

## Results and discussion

**Figure 1:**
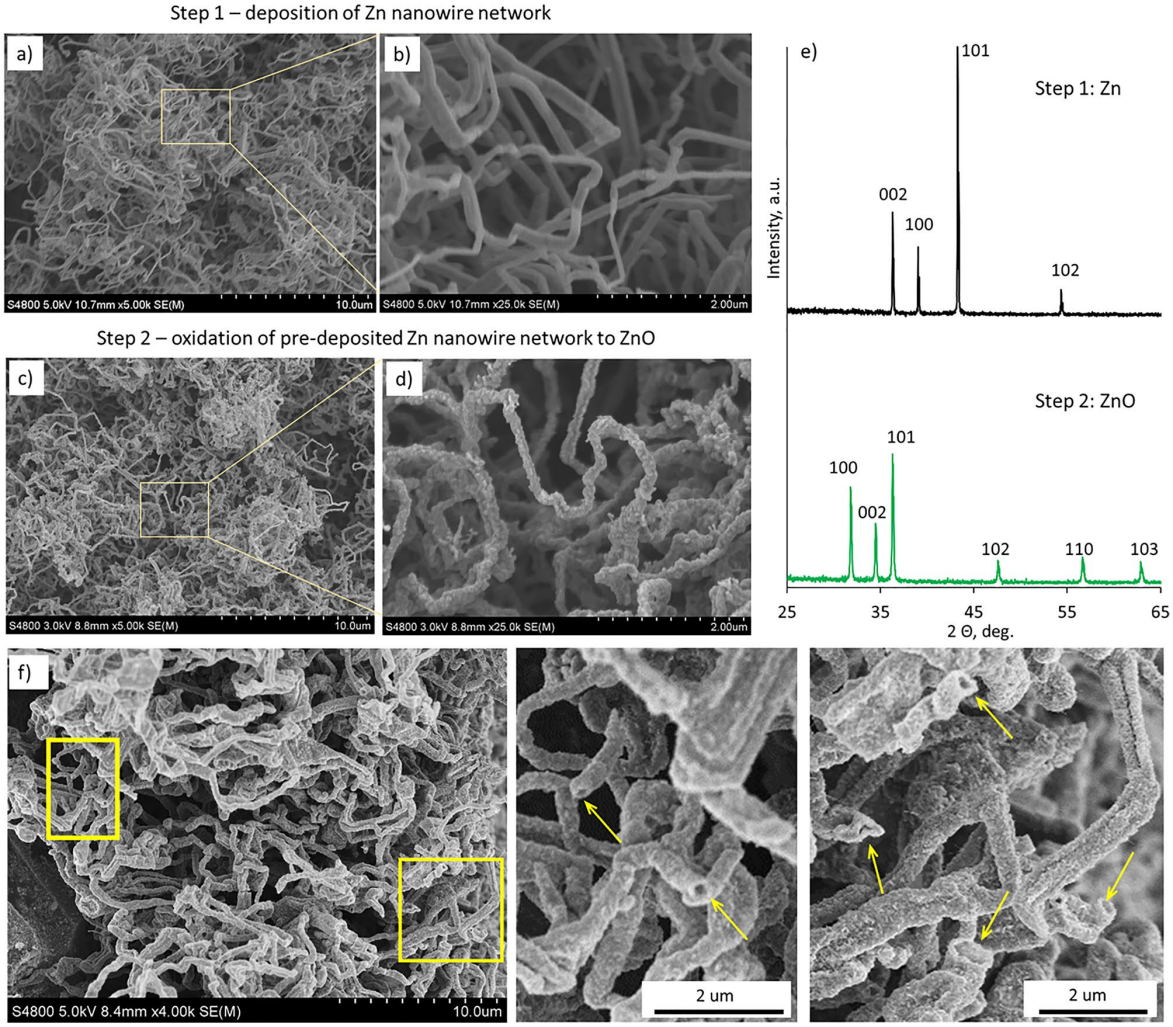
SEM images of deposited on a glass substrate Zn nanowire networks at low (**a**) and high (**b**) magnification; ZnO nanowire networks after oxidation of pre-deposited Zn nanowire networks at low (**c**) and high (**d**) magnification; XRD patterns of Zn nanowire networks (black curve) and ZnO nanowire networks (green curve); (**f**) left panel—an SEM image of the cross-section of as-grown ZnO nanowire network, revealing nanotube-like structure of the ZnO nanowires; right panel—magnified SEM images of the selected areas. Yellow arrows indicate nanowire cross-sections where tubular structure can be observed.

Deposited on a substrate, a metallic Zn nanowire network typically consists of randomly arranged interconnected nanowires of average radii 50–100 nm and length of a few μm (Fig. 1a,b). The thicknesses of the Zn nanowire network layers on glass substrates varied between 10 and 100 μm. XRD patterns obtained for the Zn nanowire network revealed diffraction peaks corresponding respectively to the (002), (100), (101), and (102) crystallographic planes of hexagonal phase Zn (Fig. 1e, black curve). The intensity of these diffraction peaks corresponds to that of the reference Zn metal, thus indicating no predominating growth direction of Zn nanowires. No zinc oxide phases were detected, thus proving that deposition conditions were optimal for forming metallic Zn nanowires. After oxidation, the oxidized Zn nanowire network changed its color from grey to white, characteristic of ZnO. XRD patterns obtained for the oxidized nanowire network revealed diffraction peaks corresponding to (100), (002), (101), (102), (110), and (103) crystallographic planes of wurtzite ZnO (Fig. 1e, green curve). No diffraction peaks corresponding to metallic Zn were detected, indicating a complete transformation of metallic Zn nanowires to ZnO. SEM inspection of the oxidized nanowire network revealed the change in the surface morphology of the nanowires from smooth to grainy (Fig. 1c,d) and the increase of radii of the nanowires from 50–100 nm to 100–200 nm. The average thickness of the ZnO nanowire network layer on a glass substrate also increased compared to the thickness of the initial Zn nanowire network by ~ 50%.

Presumably, the increase of the average radii of the ZnO nanowires and, consequently, of the total thickness of the ZnO nanowire network compared to the initial Zn nanowires may be related to the solid state up diffusion model of Zn oxidation^26^. After the initial ZnO layer is formed at the surface of the Zn nanowire, Zn^2+^ diffuses from the Zn core to the surface, driven by force generated by a contact electrical field at Zn-ZnO interface, and then forms the ZnO with O^2*–*^ in the air. The diffusion process continues until the oxidation is complete, leaving the core of the nanowire hollow. This presumption is supported by the detailed SEM inspection of the cross-section of as-grown ZnO nanowire networks, showing that the nanowires have a tubular structure (Fig. 1f). The tubular structure of the ZnO nanowires was observed throughout the cross-section of the sample; no significant differences in the morphology of the ZnO nanowires in the top and bottom layers of the network were observed. This observation is consistent with the reports on the growth of ZnO nanowires by oxidation of metallic Zn nanowires pre-fabricated by nebulized spray pyrolysis, where the transformation of smooth, fully metallic Zn nanowires to grainy ZnO nanotubes was observed^29^.

**Figure 2:**
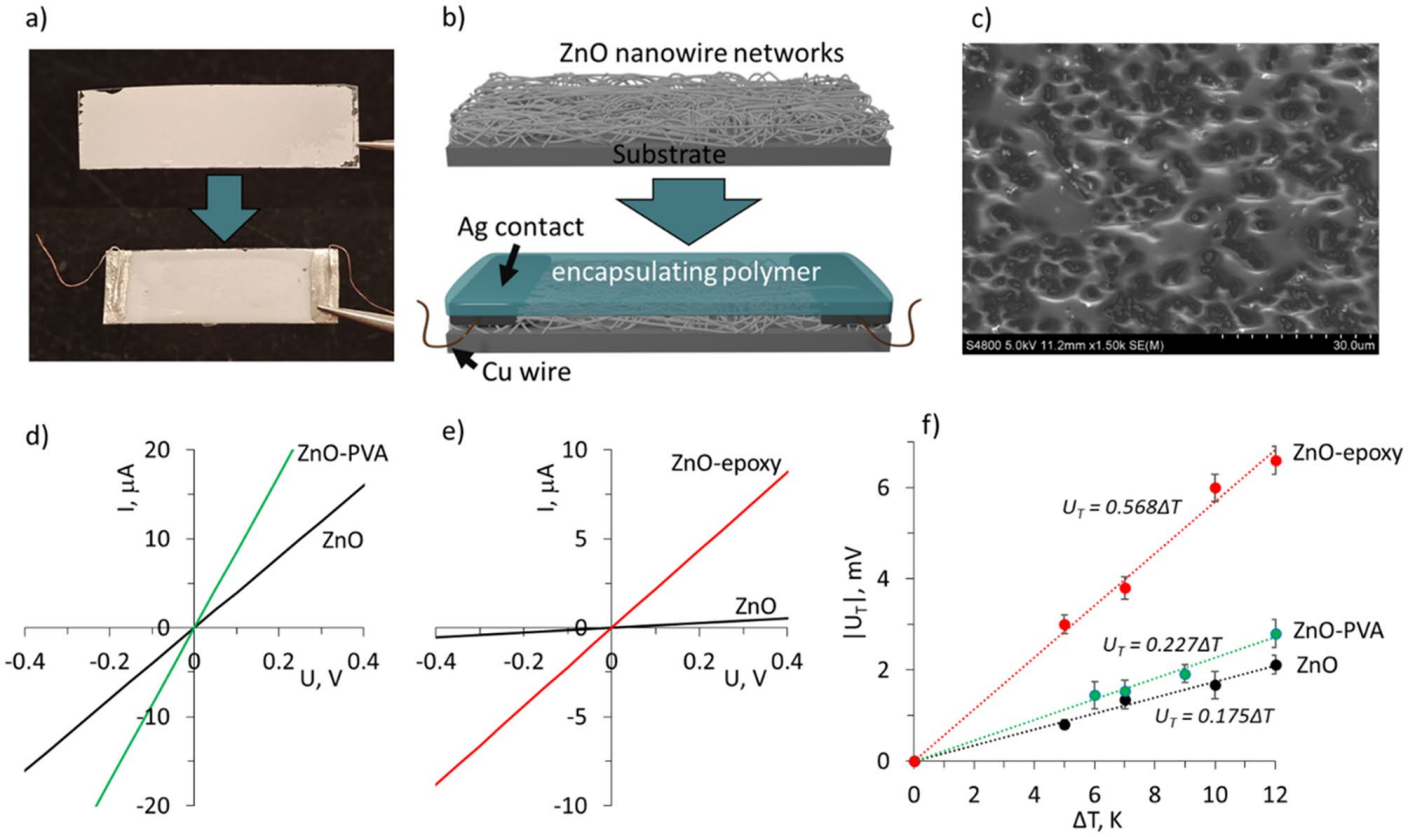
(**a**) photographs and (**b**) schematics of the encapsulation process of the ZnO nanowire networks; (**c**) scanning electron microscope image of the bottom surface of the encapsulated ZnO nanowire network; (**c**,**d**) Examples of current–voltage curves of ZnO nanowire networks: (**c**) before (black curve) and after (green curve) encapsulation in polyvinyl alcohol (PVA), and (**d**) before (black curve) and after (red curve) encapsulation in the epoxy adhesive; (**f**) absolute values of thermally generated by ZnO nanowire networks voltage U_T_ vs. temperature difference between the sides of the network: before encapsulation (black circles), after encapsulation in PVA (green circles), and after encapsulation in epoxy adhesive (red circles).

The images and schematics of the encapsulation of ZnO nanowire networks are shown in Fig. 2a and b. The drop-casting of the polymer over the ZnO nanowire network results in penetration of the polymer throughout the network to the substrate, as shown in Fig. 2c, illustrating the bottom surface of the encapsulated ZnO nanowire network. The current–voltage curves of synthesized ZnO nanowire networks before and after encapsulation showed linear behavior, indicating good electrical contacts between the nanowires throughout the nanowire network both before and after encapsulation (Fig. 2d and e).

Resistances of different samples of not-encapsulated ZnO nanowire networks were 10^4^–10^6^ Ω, depending on the linear dimensions of the samples. The estimated electrical conductivity σ of these samples was ~ 0.15 S m^−1^. Encapsulation of the ZnO nanowire networks resulted in the increase of their electrical conductivity *σ* in comparison to the initial resistance of not-encapsulated network by a factor of ~ 3 for ZnO network encapsulated in PVA (further in text referred as ZnO-PVA), and by a factor of ~ 18 for ZnO network encapsulated in epoxy adhesive (further in text referred as ZnO-epoxy), reaching 0.42 and 2.75 S m^−1^ respectively (Table 1).

**Table 1:** Electrical conductivity σ, Seebeck coefficient S, power factor PF, thermal conductivity k, and calculated figure of merit ZT of the ZnO nanowire networks before and after encapsulation, measured at room temperature under ambient conditions and comparison with the results published for other similar flexible thin films.

Sample	σ, S m^−1^	S, µV K^−1^	PF, μW m^−1^ K^−2^	k, W m^−1^ K^−1^	ZT
Bare ZnO, this work	0.15 ± 0.05	− 175 ± 10	0.0046 ± 0.0003	46*	(2.9 ± 0.1)·10^–8^
ZnO-PVA, this work	0.42 ± 0.03	− 225 ± 15	0.021 ± 0.004	75 ± 5	(8.4 ± 0.2)·10^–8^
ZnO-epoxy, this work	2.75 ± 0.05	− 570 ± 30	0.85 ± 0.05	90 ± 5	(2.9 ± 0.1)·10^–6^
ZnO thin film deposited by ALD on polyimide substrate^22^	~ 5	− 97	~ 0.06	–	–
PEDOT:PSS/ZnO hybrid composite^40^	~ 0.005	30	3.5·10^–6^	–	–
Bi_2_Se_3_-MWCNT-PVA^48^	111 ± 33	− 56 ± 1	0.4 ± 0.03	285 ± 60	(4.3 ± 0.3)·10^–9^

*Thermal conductivity data for the bare ZnO are taken from the ref.^2^.

**Figure 3:**
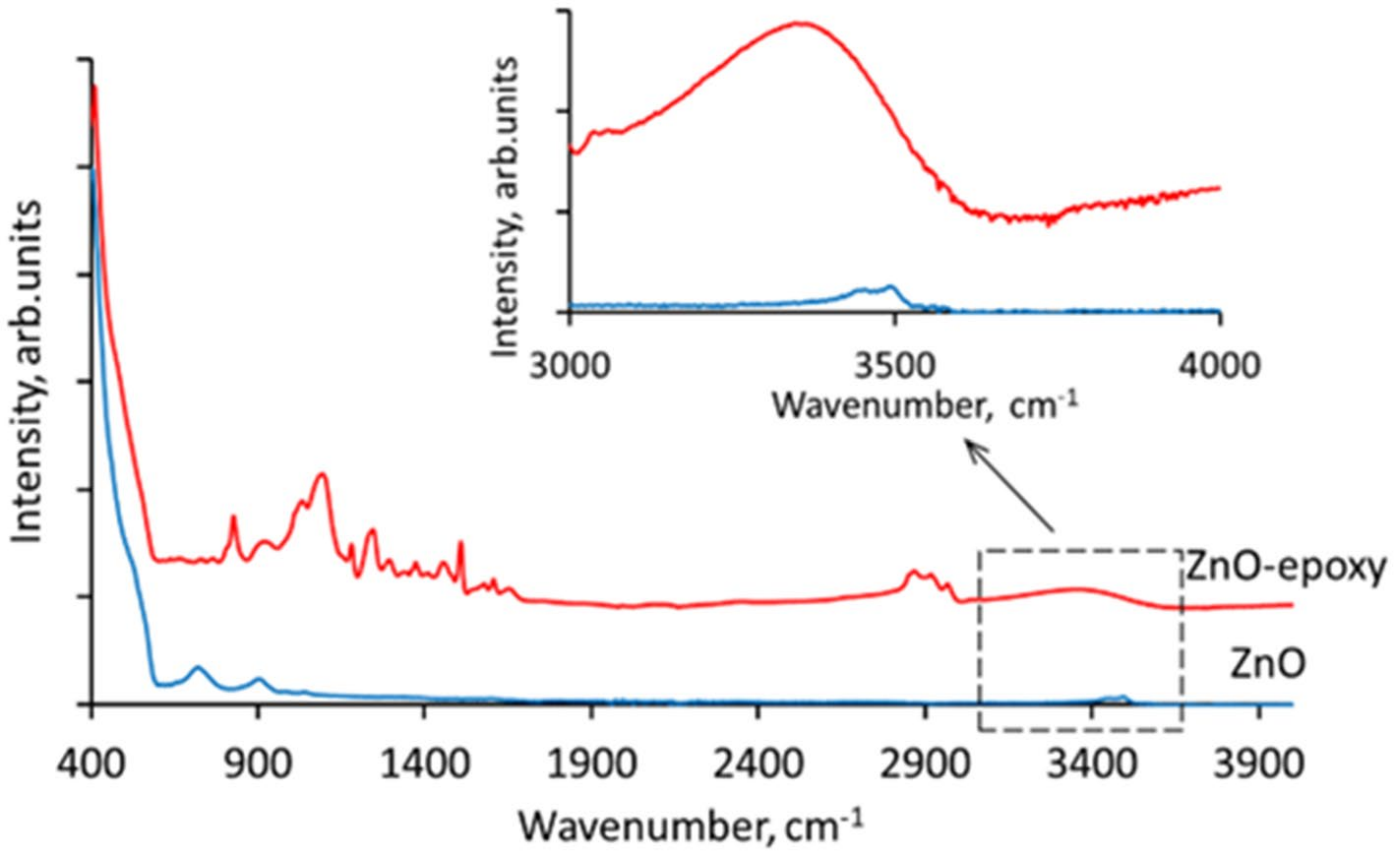
Fourier transform infrared (FTIR) spectra of bare (blue curve) and encapsulated in epoxy adhesive (red curve) ZnO nanowire networks.

Presumably, the increase in the conductivity of the encapsulated networks may be explained by the interaction between the surfaces of ZnO nanowires and the encapsulating binder. It has been shown previously that PVA/ZnO films exhibit higher electrical conductance in comparison with the bare ZnO films due to the contribution of the PVA-ZnO interface, which becomes the main conductance path for the electrons due to the chemisorption of water molecules from PVA, which can be described by the following equations^55^:

1 $$ {\text{H}}_{2} {\text{O}} + 2{\text{Zn}} + {\text{O}}_{v} \leftrightarrow \, 2{\text{Zn}}^{ + } {-}{\text{OH}} + \, V_{o} + \, 2e^{-} $$

2 $$ {\text{O}}_{v} \to V_{o} + {\text{ O}}^{{2{-}}} $$

3 $$ {\text{H}}^{ + } + {\text{ O}}^{{2{-}}} \to {\text{OH}}^{-} $$

where *O*_*v*_ is the ZnO lattice oxygen, and *V*_*o*_ is the oxygen vacancy in the ZnO lattice. Water chemisorption (Eqs. 1–3) results in the formation of hydrogen bonds with ZnO and oxygen ionization in the lattice sites of n-type ZnO followed by formation of hydroxyl (OH) groups and generation of free electrons, thus creating a conduction path along the ZnO-PVA interface^55^. Regarding the ZnO-epoxy system, it has been shown previously by the other research groups that the interaction of ZnO nanoparticles with epoxy involves the formation of hydrogen bonds between the epoxide groups and free hydroxyl (OH) groups attached to the ZnO surface or absorbed by ZnO water molecules, and results in the formation of dual nanolayer interface between the ZnO and epoxy^52,56^. Performed FTIR characterization of the ZnO and ZnO-epoxy samples revealed the presence of a low-intensity peak at approximately 3000–3500 cm^−1^ (Fig. 3), which can be attributed to the -OH absorption peak in epoxy adhesive^57^.

The first nanolayer of the interface consists of strongly bonded to each other and the ZnO surface epoxy segments. The high number of hydrogen bonds in the first ZnO-epoxy interface nanolayer mitigates the interface polarization and enhances the transport of charge carriers, making this layer electrically conductive^58^. Presumably, the mitigation of the interface polarization is the reason for the significantly higher electrical conductance of the ZnO-epoxy compared to ZnO-PVA.

Increase in the electrical conductivity of the ZnO nanowire networks after their encapsulation in PVA or epoxy adhesive was accompanied by simultaneous increase of the absolute values of the Seebeck coefficient │*S*│ by ~ 22% for ZnO-PVA (from 175 to 225 µV K^−1^, Fig. 2f, Table 1) and by ~ 225% for ZnO-epoxy (from 175 to 570  µV K^−1^, Fig. 2f, Table 1). The increase of the │*S*│ of the ZnO nanowire networks after their encapsulation in PVA may be related to the presence of oxygen vacancies and other defects formed at the surfaces of ZnO nanowires and the ZnO-PVA interface, serving as charge trapping sites. In the case of the ZnO-epoxy system, the second nanolayer of the ZnO-epoxy interface formed in the ZnO-epoxy system consists of loose epoxy segments, facilitating the easy transfer of charge carriers, as well as having a high probability of defects, which may act as charge trapping sites^58,59^. The high surface-to-volume ratio of ZnO nanowire networks and the presence of charge trapping defects at ZnO-PVA and ZnO-epoxy interfaces may result in a decrease of charge carrier concentration in the ZnO-binder system, which, in accordance with Mott’s relation between the Seebeck coefficient and charge carrier concentration would result in the increase of the Seebeck coefficient^60^:

4 $$S=\frac{8{\pi }^{2}{k}_{B}^{2}T}{3e{h}^{2}}{m}^{*}{\left(\frac{\pi }{3n}\right)}^\frac{2}{3}$$

where S is the Seebeck coefficient, *k*_*B*_ is Boltzmann’s constant, e is the electron charge, *h* is Planck’s constant, *T* is the absolute measurement temperature, m* is the effective mass of the carrier (m* = *0.23 m*_*0*_ (electron mass) for ZnO^61^), and n is charge carrier mobility. The charge carrier concentrations estimated for ZnO, ZnO-PVA, and ZnO-epoxy systems using Eq. (4) were 2.5·10^18^ cm^−3^, 1.7·10^18^ cm^−3^, and 0.4·10^18^ cm^−3^ respectively. The charge carrier mobility can be estimated by the following equation:

5 $$\mu = \frac{\sigma }{ne}$$

The calculated using Eq. (5) values of charge carrier mobility were 0.00375 cm^2^ V^−1^ s^−1^ (bare ZnO), 0.015 cm^2^ V^−1^ s^−1^ (ZnO-PVA), and 0.4 cm^2^ V^−1^ s^−1^ (ZnO-epoxy). Probably, the very low values μ estimated for bare ZnO nanowire network are related to the string scattering of the charge carriers due to the sponge-like architecture of the network, consisting of tubular nanowires with grainy structure. Encapsulation of the nanowire network in PVA and epoxy adhesive resulted in the increase of mobility of the charge carrier concentrations by ~ 4 and ~ 106 times, respectively. This result supports the hypothesis that the interface layer between the ZnO and the encapsulating polymer facilitates and enhances the transport of charge carriers.

The calculated PF of the bare ZnO nanowire network was ~ 4.6 nW m^−1^ K^−2^ at room temperature, which is insufficient for near-room temperature thermoelectric applications. However, the PFs of the PVA- and epoxy-encapsulated ZnO-nanowire networks increased by a factor of ~ 5 and ~ 185, respectively, compared to the bare ZnO networks, reaching ~ 0.85 µW m^−1^ K^−2^ for ZnO-epoxy samples (Table 1). This value was impressively higher than PF values reported by other groups for ZnO-based flexible thermoelectric films (0.06 and 3.5·10^–6^ µW m^−1^ K^−2^ respectively for ALD-deposited ZnO thin films on polyimide substrates^22^ and PEDOT:PSS/ZnO microparticle composite^40^), and twice as high compared with the state-of-the-art n-type PVA-encapsulated Bi_2_Se_3_-MWCNT flexible networks (0.4 µW m^−1^ K^−2^)^48^. The calculated ZT at room temperature of the bare ZnO, and ZnO-PVA were in the range of 10^–8^–10^–7^ (Table 1). However, due to the high Seebeck coefficient and relatively low thermal conductivity, the ZT of the ZnO-epoxy sample reached ~ 3·10^–6^, which is by 3 orders of magnitude higher than obtained for the PVA-Bi_2_Se_3_-MWCNT flexible thermoelectric films (Table 1). These results illustrate good perspectives of ZnO-epoxy composites for near-room temperature low-grade heat conversion, as well as in nano- and micro-power applications, such as, for example, environmental health monitoring.

**Figure 4:**
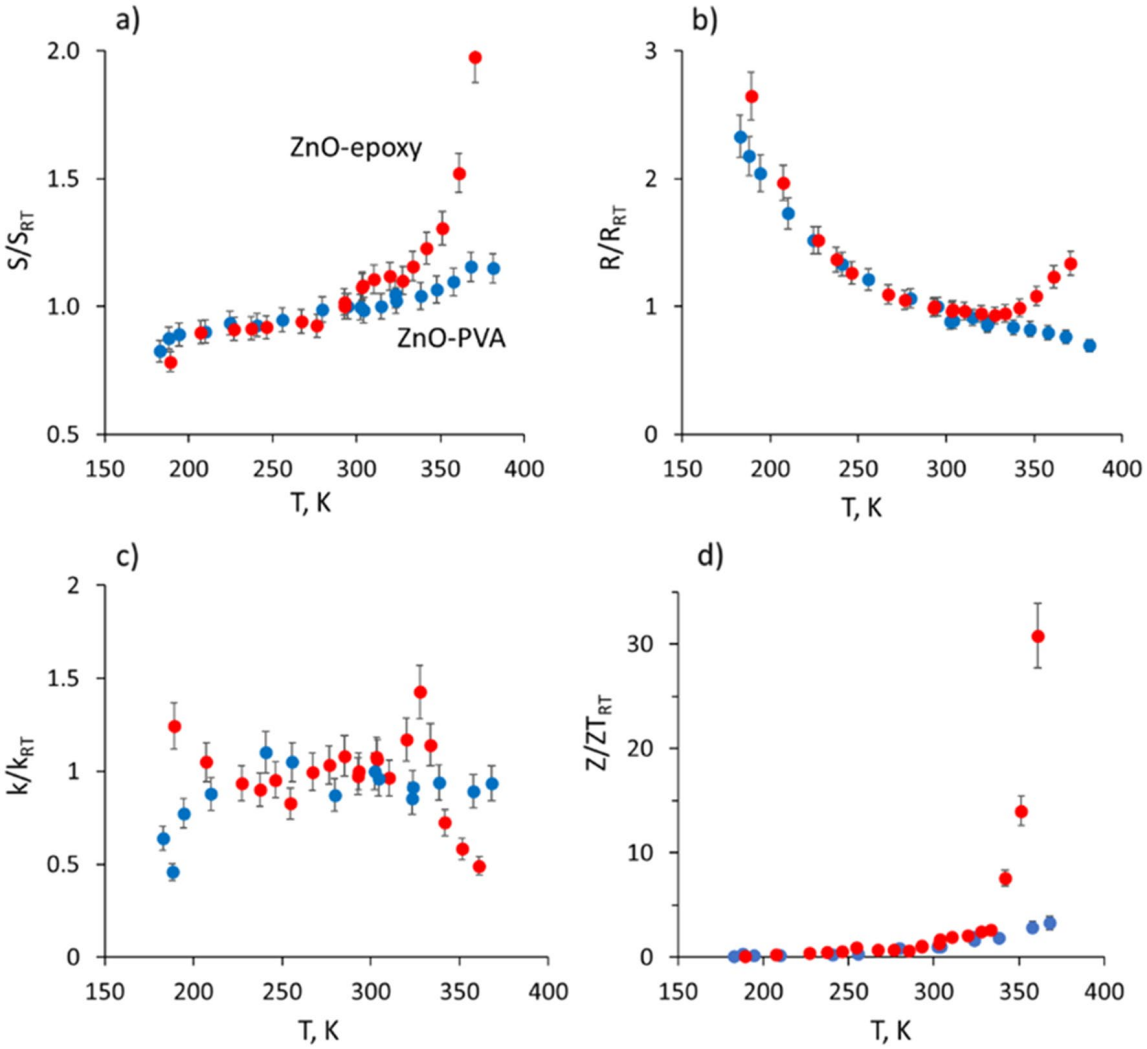
Temperature dependencies relative to the room-temperature (RT) values of (**a**) Seebeck coefficient; (**b**) resistance; (**c**) thermal conductivity; (**d**) thermoelectric figure of merit of ZnO nanowire networks encapsulated in PVA (blue dots) and epoxy adhesive (red dots).

The temperature dependencies of the thermoelectric properties of the ZnO-PVA and ZnO-epoxy samples in the target temperature range for low-grade heat conversion (180–380 K) showed that Seebeci coefficient of ZnO-PVA sample was relatively stable in this range, showing gradual decrease down to 80% of the room-temperature value (S_RT_) with the decrease of temperature down to 180 K, and increase up to 115% of the S_RT_ with the increase of temperature up to 380 K (Fig. 4a, blue dots).

The temperature increase of the Seebeck coefficient of ZnO-PVA samples was accompanied by a decrease in the resistance, which was expected for semiconductor behavior (Fig. 4b, blue dots). With the temperature increase from 180 to 380 K, the resistance of the ZnO-PVA sample decreased from ~ 230% of room-temperature value R_RT_ to ~ 70% (Fig. 4b). In contrast, ZnO-epoxy showed similar to ZnO-PVA sample behavior of the Seebeck coefficient and resistance at temperatures below 300 K (Fig. 4b, red dots). However, with the temperature increase above 300 K, the Seebeck coefficient of the ZnO-epoxy sample showed a rapid increase, reaching ~ 200% from the S_RT_ at 370 K (Fig. 4a, red dots). Such increase in the Seebeck coefficient was accompanied by the U-torn of the resistance of the ZnO-epoxy sample at temperatures above 300 K, reaching ~ 135% of the R_RT_ at 370 K. Presumably, such behavior of the ZnO-epoxy sample at temperatures above 300 K was related to the changes in the ZnO-epoxy interface, for example, to the temperature-promoted activation of charge traps when the temperature approaches the operational limit of the epoxy adhesive and it starts transition to a viscous state, resulting in changes in charge carrier concentration or charge carrier filtering at the ZnO-epoxy interface, and further increase of the Seebeck coefficient. In turn, the thermal conductivity of ZnO-PVA and ZnO-epoxy did not show pronounced dependence on temperature in the temperature range 200–330 K (Fig. 4c). However, ZnO-PVA samples showed up to 50% lower compared to the room-temperature value k_RT_ thermal conductivity in the temperature range 180–200 K, which may be related to the partial freezing of the PVA (Fig. 4c, blue dots). In contrast, ZnO-epoxy samples showed lower up to 50% thermal conductivity at temperatures around and above 350 K, which may be related to the changes in the epoxy structure at elevated temperatures (for example, partial transition to a viscous state). Temperature dependencies of the ZT of ZnO-PVA and ZnO-epoxy samples showed a gradual increase of the ZT of ZnO-PVA with the increase of temperature, reaching ~ 3.3 higher ZT at 380 K compared to its room-temperature value Z_RT_ (Fig. 4d, blue dots). In contrast, ZT of ZnO-epoxy showed a rapid increase at temperatures above 330 K, reaching ~ 30 times higher ZT value at temperature 370 K compared to the Z_RT_ (Fig. 4d, red dots). These results suggest epoxy-encapsulated ZnO nanowire networks for low-grade waste heat conversion applications in the temperature range 330–370 K, for example, from hot pipes or other similar heated surfaces.

**Figure 5:**
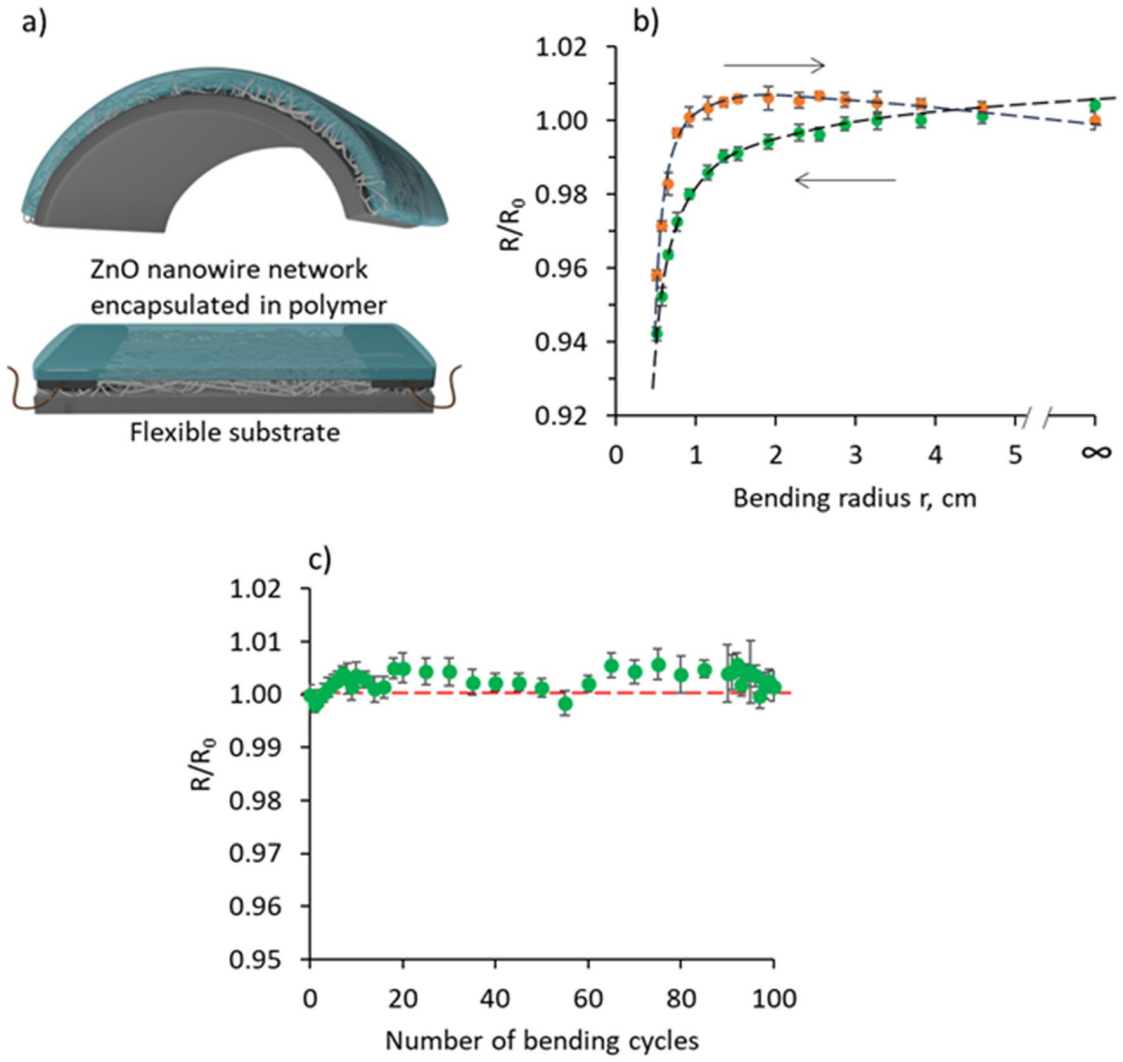
(**a**) A schematic indicating the bending direction of the PVA-encapsulated ZnO networks. (**b**,**c**) relative changes in the electrical resistance of the PVA-encapsulated ZnO networks vs (**b**) its bending radius and (**c**) consecutive bending cycles. R is the resistance of the bent sample, and R_0_ is the initial resistance of the not-bent sample.

In addition, bending tests performed for PVA-encapsulated ZnO nanowire samples (Fig. 5a) showed that the resistance of the sample tends to decrease reversibly with the decrease of the bending radius (Fig. 5b). Presumably, the decrease in the resistance is related to the increased number of direct mechanical and electrical contacts between the ZnO nanowires when bent, resulting in an increase in the effective cross-section of the sample. More rapid return to the initial resistance values during unbending of the sample may be related to the elastic properties of the PVA as well as to the changes in the electrical contacts within the ZnO nanowire network during the bending, i.e., destruction of several contacts and formation of a new ones. The total decrease in the resistance of the sample was within 6% from the initial resistance *R*_*0*_ of the not-bent sample (Fig. 5b).

ZnO-PVA encapsulated samples also showed very good stability during 100 consequent bending cycles down to a 5 mm radius (Fig. 5c). The resistance of the sample showed an insignificant increase within 0.5% from the initial resistance value R_0_ of the not-bent sample during the first ten bending cycles, followed by stabilization of the resistance within 1% from R_0_ for the subsequent 90 bending cycles.

## Conclusions

Self-assembling ZnO nanowire networks were fabricated by thermal oxidation in air pre-deposited by thermal evaporation metallic Zn nanowire network. XRD studies of the oxidized Zn nanowire networks proved a complete transition of metallic Zn to ZnO. At the same time, an in-depth SEM investigation revealed that the resulting ZnO nanowires have tubular structure, presumably resulting from the diffusion of Zn^2+^ from the core of the nanowire to the surface during the oxidation process. As-grown ZnO nanowire networks showed electrical conductivity of approximately 0.15 S m^−1^ and Seebeck coefficients of ~ *–*175 µV K^−1^, which is comparable with some theoretical and experimental Seebeck coefficient values reported for ZnO nanowires obtained by thermal evaporation of ZnO (~  − 110 to 190 µV K^−1^). After the encapsulation in polyvinyl alcohol (PVA) and commercially available epoxy adhesive, ZnO nanowire networks showed a significant increase in the electrical conductivity by a factor of ~ 3 and ~ 18 for ZnO-PVA and ZnO-epoxy systems, respectively, reaching the value of 2.75 S m^−1^ for the epoxy-encapsulated sample. The increase in electrical conductivity was attributed to the formation of electrically conductive channels at the ZnO-encapsulating polymer interface. The increase in the electrical conductivity after the encapsulation of the ZnO nanowire networks was accompanied by the increase in the Seebeck coefficient of ZnO-PVA and ZnO-epoxy systems in comparison with not encapsulated ZnO nanowire networks by ~ 22% and ~ 225% for ZnO-PVA and ZnO-epoxy systems respectively, which was attributed to the changes in charge carrier concentrations in these systems caused by the presence of oxygen vacancies on the surface of ZnO, as well as defect sites at the ZnO-polymer interface, which may serve as charge traps. The absolute value of the Seebeck coefficient of the ZnO-epoxy sample, measured at room temperature, reached ~ − 570 µV K^−1^, which is comparable to the best values of the room-temperature Seebeck coefficient reported for ZnO powders-based densified ceramics (~ − 600 µV K^−1^) and is significantly higher compared to the Seebeck coefficients reported for the vast majority of the state-of-the-art ZnO-based thermoelectric materials and ceramic composites, measured at elevated (500–1000 °C) temperatures. The maximal power factor and ZT at room temperature were shown by the epoxy-encapsulated ZnO nanowire networks and reached 0.85 µW m^−1^ K^−2^ and ~ 3·10^–6^, respectively, which significantly exceeded the power factor values reported previously for similar (ZnO- and/or non-conductive polymers-based) flexible thermoelectric materials. A study of the temperature dependencies of the thermoelectric properties of the encapsulated ZnO nanowire networks in the target temperature range of 180–380 K showed that ZT of these materials can be improved by a factor of ~ 3 and ~ 30 for ZnO-PVA and ZnO-epoxy samples respectively with the increase of temperature up to 350–380 K. In addition, bending tests of the ZnO-PVA samples showed a reversible decrease in the resistance of the sample up to ~ 6% from the resistance value of the unbent sample when bent down to a 5 mm radius, which was attributed to the formation/destruction of new direct electrical connections between the ZnO nanowires during the bending. ZnO-PVA samples also showed very good mechanical and electrical stability during 100 consecutive bending cycles down to a 5 mm radius. The fluctuations of the resistance of the sample during the repetitive bending tests did not exceed 1% from the initial resistance of the sample. The simplicity of fabrication, flexibility, and significantly enhanced thermoelectric properties of PVA- and especially epoxy-encapsulated self-assembling ZnO nanowire networks open a new path for applying such systems in near-room temperature flexible thermoelectrics for low-grade heat conversion.

## Data Availability

The data presented in this study are available on request from the corresponding author. The data are not publicly available as they are part of ongoing research.
